# Prevalence of Noncommunicable Diseases in Japan Using a Newly Developed Administrative Claims Database Covering Young, Middle-aged, and Elderly People

**DOI:** 10.31662/jmaj.2021-0189

**Published:** 2022-02-28

**Authors:** Akira Okada, Hideo Yasunaga

**Affiliations:** 1 Department of Prevention of Diabetes and Lifestyle-Related Diseases, Graduate School of Medicine, The University of Tokyo, Tokyo, Japan; 2 Department of Clinical Epidemiology and Health Economics, The University of Tokyo, Tokyo, Japan

**Keywords:** Claims database, health insurance, epidemiology, Japan, elderly population

## Abstract

**Introduction:**

Noncommunicable diseases (NCDs) are an ongoing public health problem globally. The present study aimed to estimate the prevalence of NCDs in Japan using a newly developed, commercially available administrative claims database covering young, middle-aged, and elderly people.

**Methods:**

We compared the age-stratified population distribution between the DeSC administrative claims database and the population estimates. We calculated the 1 year prevalence of several NCDs using the DeSC database and compared the prevalence of diabetes mellitus and hypertension between the DeSC database and the Japan National Health and Nutrition Survey.

**Results:**

The age distribution of the population included in the DeSC database was similar to that of the population estimates. The estimated prevalence rates were as follows: diabetes mellitus (12.2%), hypertension (20.9%), ischemic heart disease (5.6%), heart failure (5.3%), cerebral infarction (3.4%), stroke (3.7%), gastric cancer (0.6%), colorectal cancer (0.8%), breast cancer (1.5%), prostate cancer (0.6%), cataract (7.1%), depression (3.5%), and osteoporosis (6.3%). The estimated prevalence of diabetes and hypertension was comparable with that of the National Health and Nutrition Survey.

**Conclusions:**

The distribution of age and sex in the database was comparable with that of the population estimates. The prevalence of diabetes mellitus and hypertension was comparable with that in a previously reported national survey. Our data can be utilized as basic information for policymaking in clinical medicine and public health in Japan.

## Introduction

Noncommunicable diseases (NCDs) such as diabetes mellitus, heart disease, and cancer account for up to 70% of deaths globally ^(1)^ and thus are an ongoing public health issue in developed and developing countries ^(1), (2)^. In Japan, more than half of deaths are attributed to NCDs ^(3)^. To develop an effective health policy for preventing NCDs and reducing deaths from NCDs, it is imperative to estimate the prevalence of NCDs.

In Japan, there are several publicly available data sources for estimating the prevalence of NCDs. The Japan National Health and Nutrition Survey (NHNS), a national sampling survey, provides an estimated prevalence of diabetes and hypertension ^(4)^. The National Database of Health Insurance Claims and Specific Health Checkups of Japan (NDB) Open Data provide information on medical procedures for all inpatients and outpatients in Japan ^(5)^.

On the other hand, commercially available administrative claims databases have been established in Japan and are expected to be utilized for clinical and epidemiological studies. However, currently used commercially available claims databases may lack population representativeness because the data are collected from a single type of health insurer, including the health insurance for employees of large companies (Kempo), Japan Health Insurance Association for employees of medium and small companies (Kyokai Kempo), the National Health Insurance for nonemployees (Kokuho), and the Advanced Elderly Medical Service System for elderly people aged ≥ 75 years [Koki Koreisha Iryo Seido]. It is important to verify to what extent such databases are representative of the whole population in Japan by examining the distribution of the backgrounds (including age, sex, and diseases) before using them in epidemiological and clinical studies.

In 2021, DeSC Inc. developed a new administrative claims database, the DeSC database, which covers young, middle-aged, and elderly people from Kempo, Kokuho, and Koki Koreisha Iryo Seido. The present study utilized the DeSC database to estimate the prevalence of several NCDs (including lifestyle-related diseases, mental disorders, and cancer) to confirm the population representativeness of the database. We compared the estimated prevalence with that reported in the currently available national data.

## Materials and Methods

### Japanese universal healthcare insurance system

Since the Japanese government established the universal healthcare insurance system in 1961, virtually all of those living in Japan have been supposed to be covered by one of several public insurance systems ^(6)^. Kokuho indicates the National Health Insurance for self-employed individuals, retired individuals, and their dependents. Kempo indicates association/union-administered health insurance for salaried employees in large companies. Kyokai Kempo indicates that the Japan Health Insurance Association administered health insurance to salaried employees in medium and small companies. Kyosai Kumiai is a Mutual Aid Association that administered health insurance for other employees, including civil servants. Koki Koreisha Iryo Seido indicates the Advanced Elderly Medical Service System for all people aged 75 years or older. Specific kinds of people (e.g., those aged 65-74 years with disability certificates) can be insured by this system. As of 2019, the number of participants in Kokuho, Kempo, Kyokai Kempo, Kyosai Kumiai, and Koki Koreisha Iryo Seido were 28.7, 29.5, 38.9, 8.6, and 17.2 million people in Japan, respectively. The total number of participants in the employees’ insurance systems (Kempo, Kyokai Kempo, and Kyosai Kumiai) was 77.0 million.

### DeSC database

The data source of this study comprised claims data and medical checkup data provided from DeSC Healthcare Inc. Tokyo, Japan (https://desc-hc.co.jp/company). DeSC Healthcare Inc. owns epidemiological claims data of 3,000,000 insurance subscribers. This data source is anonymously processed information created for the different purposes of this study on the basis of claims data and medical checkup data provided by Kokuho, Kempo, and Koki Koreisha Iryo Seido.

For the claims data, all medical or dental diagnoses were recorded according to the International Classification of Diseases 10th Revision (ICD-10) codes and recorded in Japanese free texts. The DeSC database also contains information on medications prescribed by medical doctors or dentists and costs reimbursed for examinations, procedures, surgery, and anesthesia. Drug specifications were also recorded on the basis of anatomical therapeutic chemical classification systems based on the World Health Organization or the European Pharmaceutical Market Research Association. The number of days prescribed for each prescribed drug was also recorded.

The DeSC database also contains information on annual health checkups in approximately 30% of the entire population in the database. The health checkup data included information on height, weight, and blood pressure measurements, clinical laboratory tests (e.g., complete blood count, and biochemistry), and questionnaires on lifestyle factors (e.g., smoking and alcohol habits, medical history of stroke, or ischemic heart disease). Body mass index was calculated as weight (kg) divided by the square of the height (m).

### Study design and population

This was a descriptive study using the DeSC database. We extracted data from December 2019 to November 2020 on all patients in the DeSC database to show the age distribution and to estimate the prevalence of NCDs. We identified patients with specific NCDs on the basis of the following ICD-10 codes: diabetes mellitus (E10-14); hypertension (I10-15); ischemic heart disease (I20-25); heart failure (I50); cerebral infarction (I63); stroke (I60-63), cancers of the stomach (C16), colon or rectum (C18-20), breast (C50), and prostate (C61); depression (F32); dementia (I63); cataract (H25, H26, and H28); and osteoporosis (M80 or M81).

### Statistics

First, we described the distribution of age and type of insurance included in the DeSC database. To confirm whether the age distribution in the DeSC database was similar to that in the whole population of Japan, we also described the age distribution of the entire Japanese population recorded in the population estimates in 2019 ^(7)^.

Second, we calculated the 1 year prevalence of the above-mentioned NCDs. Each prevalence was stratified by the 5 year age category and sex (except prostate cancer). We referred to the reported prevalence of diabetes mellitus and hypertension using the NHNS 2019 and compared the estimated prevalence between our data and the NHNS data. The NHNS reported the prevalence of diabetes mellitus in two categories, including “strongly suspected diabetes mellitus” and “possibly suspected diabetes mellitus,” and the prevalence of hypertension as “hypertension on medication” and “hypertension without medication.”

Third, we compared the prevalence of depression and breast cancer between the Kempo and Kokuho populations. Because depression may induce sickness absence and retirement ^(8)^, we hypothesized that the prevalence of depression in the Kokuho population may be higher than that in the Kempo population. Previous studies have suggested that working conditions and socioeconomic status affect the proportion of patients undergoing cancer screening compared with the Kempo population ^(9)^. Hence, we hypothesized that the prevalence of breast cancer may differ between the Kempo and Kokuho populations.

Fourth, we calculated the proportions of patients undergoing gastrectomy (Japanese procedure codes: K655, K655-2, K655-4, K655-5, K657, and K657-2), stratified by age and sex. We referred to the reported data on the proportions of patients undergoing gastrectomy in the NDB Open Data 2019 and compared the proportions between our data and the NDB Open Data.

Lastly, we described the distribution of body mass index stratified by age and sex using health checkup data in the DeSC database. Body mass index was categorized into three groups: low (<18.5 kg/m^2^), normal (18.5 kg/m^2^ to < 25.0 kg/m^2^), and high (≥ 25.0 kg/m^2^). We referred to the reported data on the distribution of body mass index in the NHNS 2019 and compared the distribution of body mass index between our data and the NHNS data.

Because of the large sample size, we did not perform statistical testing.

### Ethical considerations

The Institutional Review Board of the Graduate School of Medicine at the University of Tokyo approved the study protocol (approval code: 2021010NI). The requirement for informed consent was waived because of the anonymous nature of the data.

Because this study used “existing anonymously processed information,” this study does not need to comply with the “Outline of ethical guidelines for medical and health research involving human subjects” (22/12/2014, Ministry of Education, Culture, Sport, Science and Technology and Ministry of Health, Labour and Welfare. Revised on 28/2/2017). There are no limitations in the utilization purpose for anonymously processed data other than that based on the Act on the Protection of Personal Information; therefore, this research can apply the anonymously processed information for different purposes from the creation with no limitations.

## Results

### Population of the DeSC database

We identified 2,220,702 individuals during the study period in the DeSC database, including 824,516 in Kempo, 1,095,713 in Kokuho, and 300,473 in Koki Koreisha Iryo Seido.

**Figure 1.**
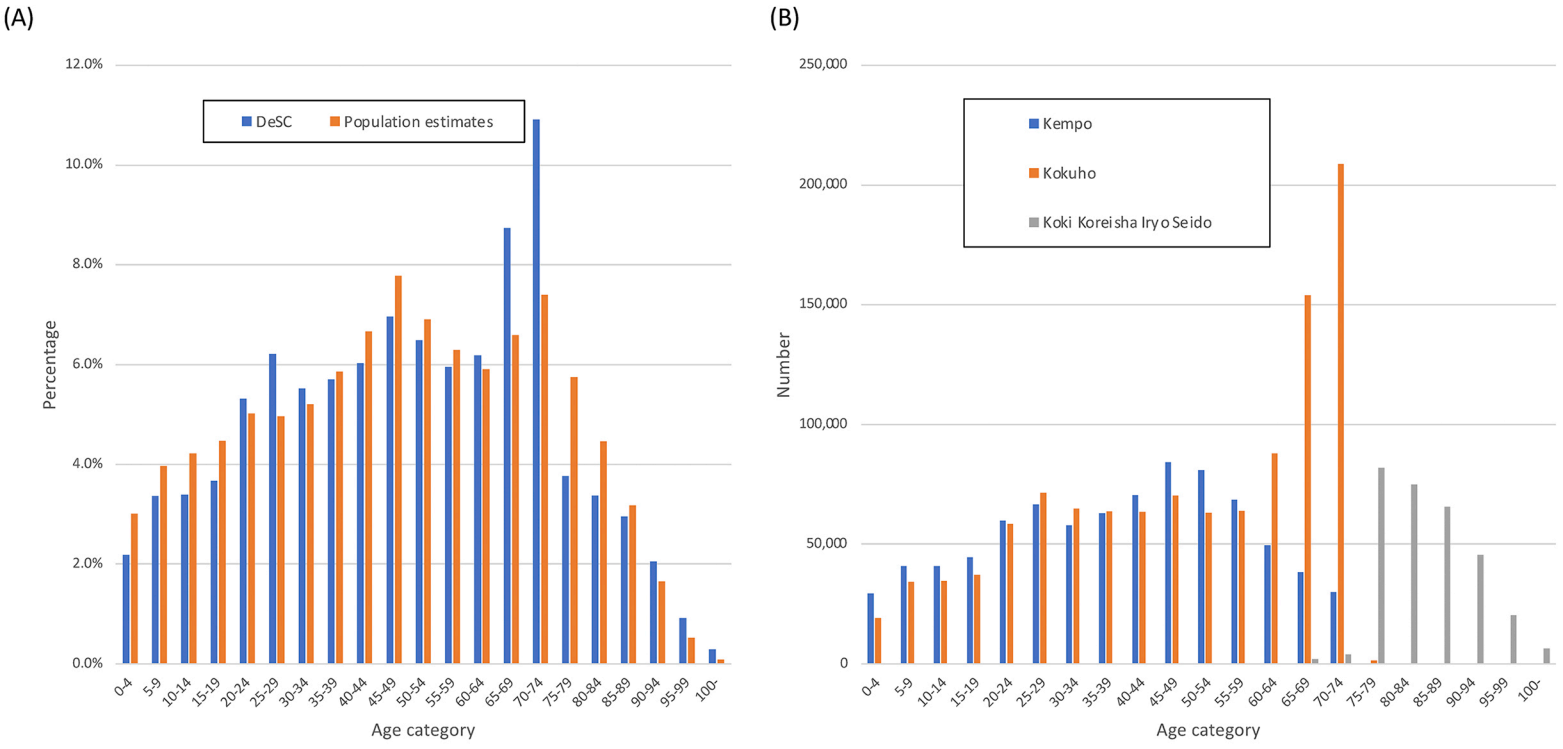
Age distribution of the population included in the DeSC database and population estimates in 2019 (A) The proportions of each age category in the DeSC database and the population estimates in 2019 (B) The numbers in each age category stratified by the three insurance systems in the DeSC database DeSC, the DeSC database; population estimates, population estimates in 2019.

Table 1 shows the proportions of those in each age category divided by insurance type in the DeSC database. Supplementary Table 1 shows the proportions of those with health checkup data by age category and by insurance type in the DeSC database. Figure 1A shows the proportions of each age category in the DeSC database and the population estimates in 2019. Figure 1B shows the numbers in each age category stratified by the three insurance systems in the DeSC database. Overall, the age distributions were similar except for higher proportions in their 60s and 70s in the DeSC database, most of whom belonged to Kokuho.

**Table 1.** Age Distribution of Those with Each Insurance.

Age category (years)	Total	Kempo	Kokuho	Koki Koreisha Iryo Seido
	N = 2,220,702	N = 824,516	N = 1,095,713	N = 300,473
0-4	2.2%	3.6%	1.7%	0.0%
5-9	3.4%	4.9%	3.1%	0.0%
10-14	3.4%	4.9%	3.1%	0.0%
15-19	3.7%	5.4%	3.4%	0.0%
20-24	5.3%	7.3%	5.3%	0.0%
25-29	6.2%	8.1%	6.5%	0.0%
30-34	5.5%	7.0%	5.9%	0.0%
35-39	5.7%	7.6%	5.8%	0.0%
40-44	6.0%	8.5%	5.8%	0.0%
45-49	7.0%	10.2%	6.4%	0.0%
50-54	6.5%	9.8%	5.8%	0.0%
55-59	6.0%	8.3%	5.8%	0.0%
60-64	6.2%	6.0%	8.0%	0.0%
65-69	8.7%	4.6%	14.1%	0.6%
70-74	10.9%	3.6%	19.1%	1.3%
75-79	3.8%	0.0%	0.1%	27.3%
80-84	3.4%	0.0%	0.0%	25.0%
85-89	3.0%	0.0%	0.0%	21.9%
90-94	2.0%	0.0%	0.0%	15.1%
95-99	0.9%	0.0%	0.0%	6.7%
≥100	0.3%	0.0%	0.0%	2.1%

### Prevalence of NCDs

After excluding those whose insurance initiation was after December 2019 and excluding those whose insurance termination was before November 2020, we obtained 1,932,021 people including 958,159 women and 973,862 men.

**Figure 2.**
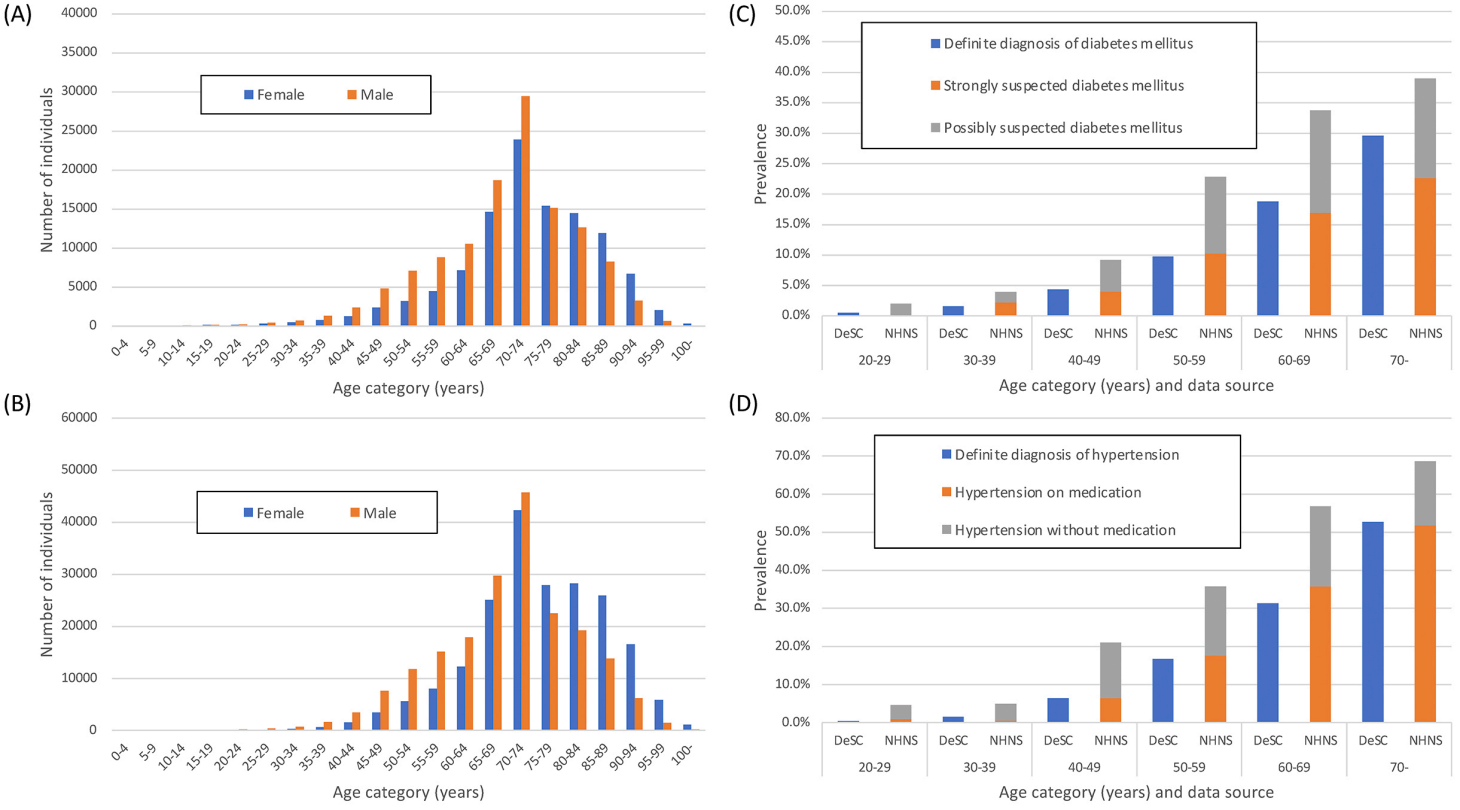
Number and prevalence of diabetes or hypertension using DeSC database and National Health and Nutrition Survey 2019 (A) Number of patients with diabetes mellitus in the DeSC database (B) Number of patients with hypertension in the DeSC database (C) Prevalence of diabetes mellitus in the DeSC database and in the NHNS 2019 (D) Prevalence of hypertension in the DeSC database and in the NHNS 2019 DeSC, the DeSC database; NHNS, National Health and Nutrition Survey 2019.

In the DeSC database, the number of patients with diabetes and hypertension was 235,089 and 402,941, respectively, and the overall prevalence of diabetes and hypertension was 12.2% and 20.9%, respectively. The prevalence of diabetes mellitus in the DeSC database was higher than the “strongly suspected diabetes mellitus” and lower than “strongly suspected diabetes mellitus” plus “possibly suspected diabetes mellitus” in the NHNS data (Figure 2A and 2C). By contrast, the prevalence of hypertension in the DeSC database was similar to the prevalence of “hypertension on medication” in the NHNS data (Figure 2B and Figure 2D).

**Figure 3.**
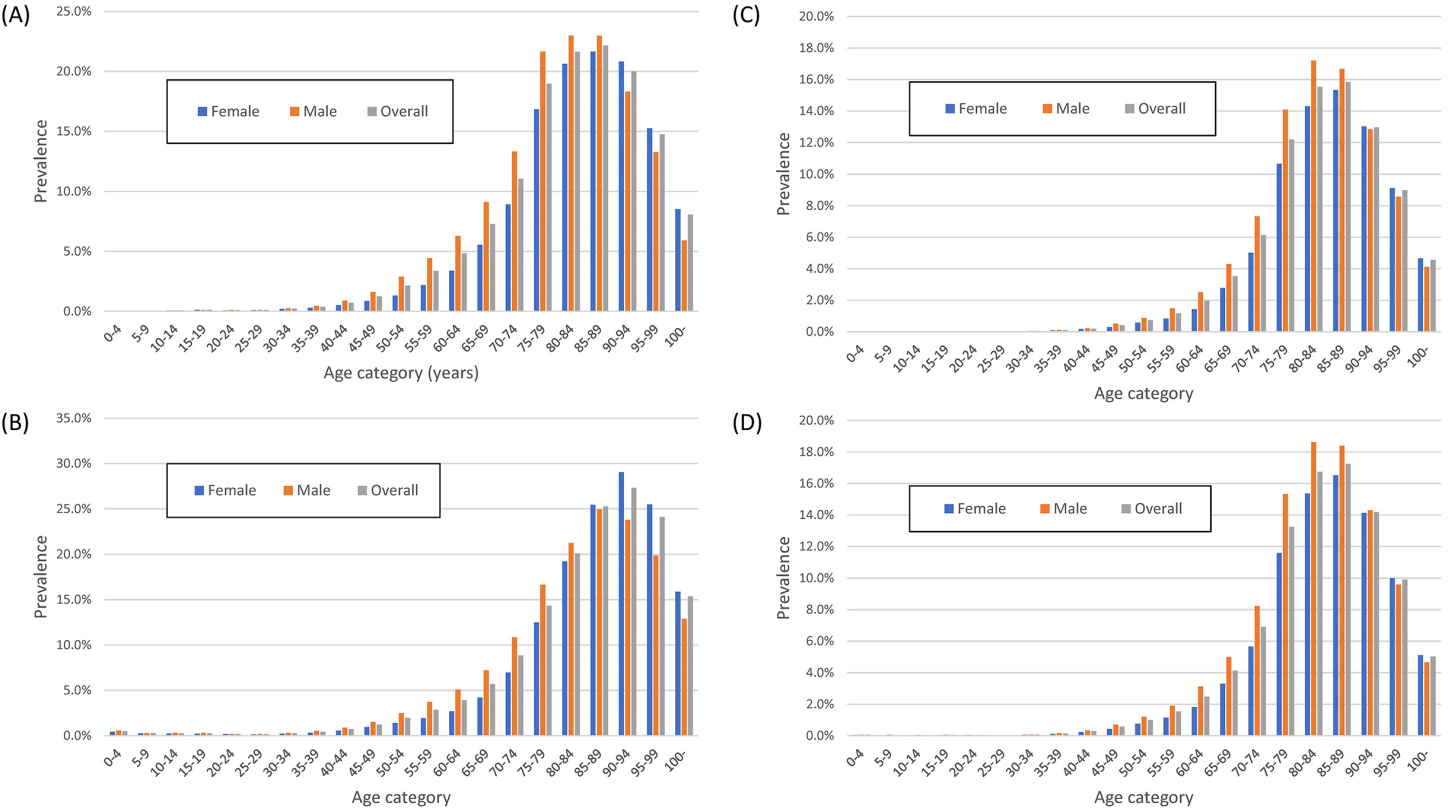
Prevalence of ischemic heart disease, heart failure, cerebral infarction, and stroke in the DeSC database (A) Prevalence of ischemic heart disease (B) Prevalence of heart failure (C) Prevalence of cerebral infarction (D) Prevalence of stroke.

The overall prevalence of ischemic heart disease, heart failure, cerebral infarction, and stroke in the DeSC database was 5.6%, 5.3%, 3.3%, and 3.7%, respectively. The prevalence of these four cardiovascular diseases was similar and peaked in the 80-to-89-year-old category (Figure 3).

**Figure 4.**
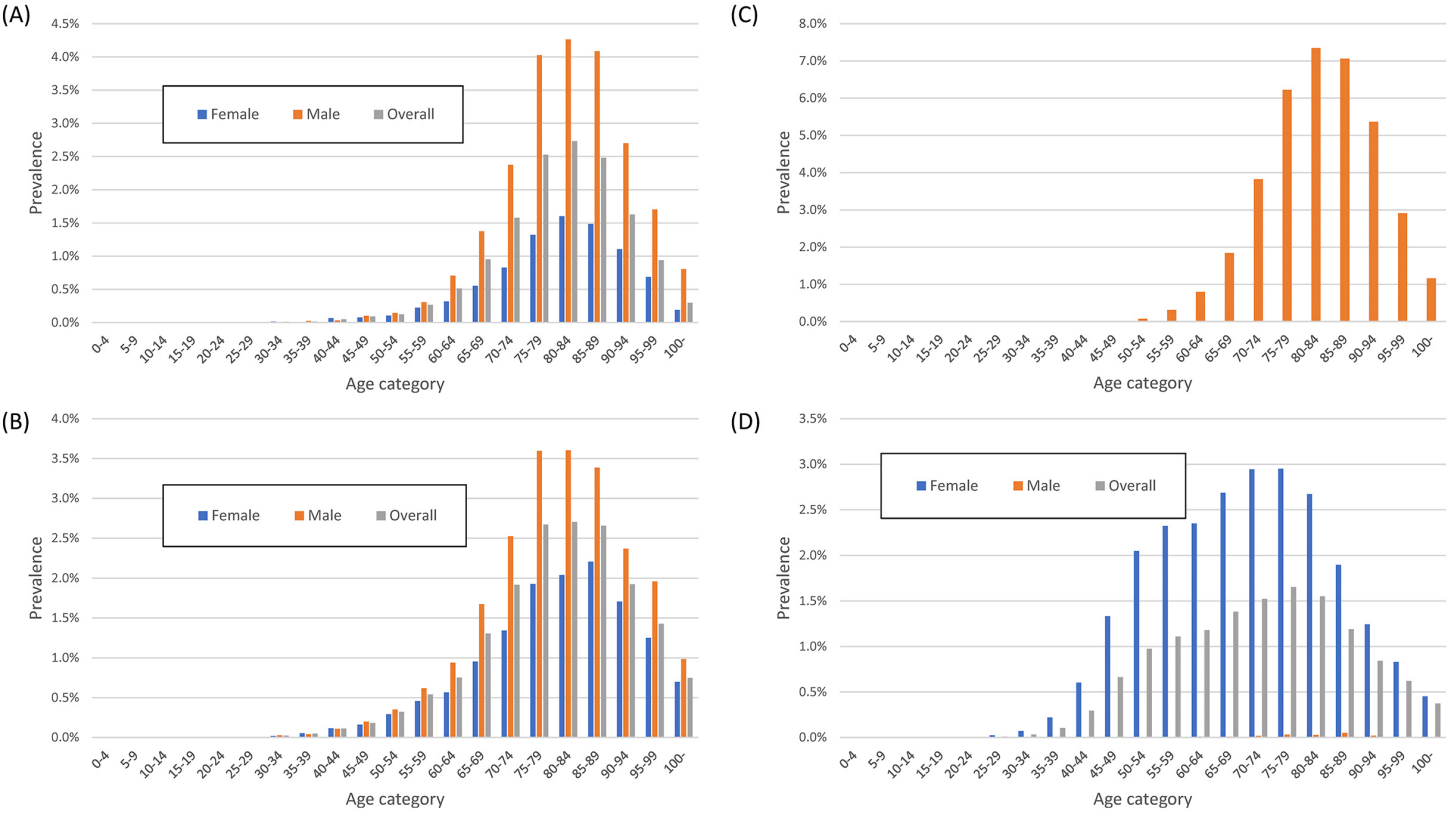
Prevalence of gastric cancer, colorectal cancer, breast cancer, and prostate cancer in the DeSC database (A) Prevalence of gastric cancer (B) Prevalence of colorectal cancer (C) Prevalence of prostate cancer in men (D) Prevalence of breast cancer.

Figure 4 shows the age-stratified prevalence of the four cancers. The overall prevalence of gastric cancer, colorectal cancer, and prostate cancer in men and breast cancer in women or men in the DeSC database was 0.6%, 0.8%, 0.6%, 1.5%, and 0.007%, respectively. The age distribution of those with either of these cancers was similar, except for breast cancer.

**Figure 5.**
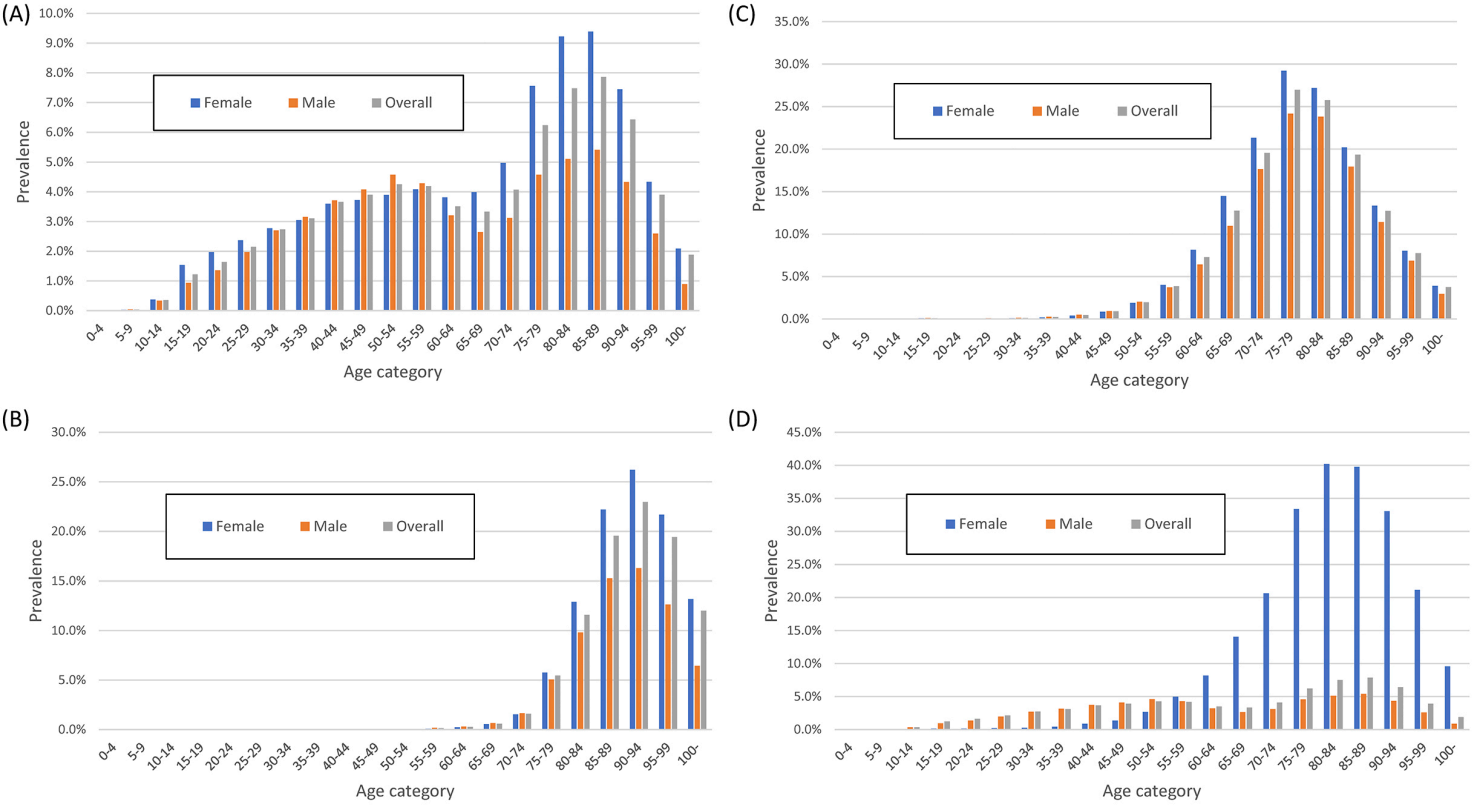
Prevalence of patients with depression, dementia, cataract, and osteoporosis in the DeSC database (A) Prevalence of depression (B) Prevalence of dementia (C) Prevalence of cataracts (D) Prevalence of osteoporosis.

Figure 5 shows the age-stratified prevalence of depression, dementia, cataracts, and osteoporosis. The overall prevalence of depression, dementia, cataracts, and osteoporosis in the DeSC database was 3.5%, 2.4%, 7.1%, and 6.3%, respectively. The prevalence of depression had bimodal peaks at 50-54 and 85-89 years. The prevalence of dementia peaked at 90-94 years. Although the prevalence of cataracts did not differ by sex, the prevalence of osteoporosis was higher in women.

Supplementary Figure 1 shows the prevalence of depression and breast cancer in those insured with Kempo or Kokuho. The prevalence of depression was comparable between Kempo and Kokuho at 50-64 years of age, whereas it was higher in Kokuho at 30-49 years of age. The prevalence of breast cancer was higher in Kokuho.

### Proportions of patients undergoing gastrectomy

**Figure 6.**
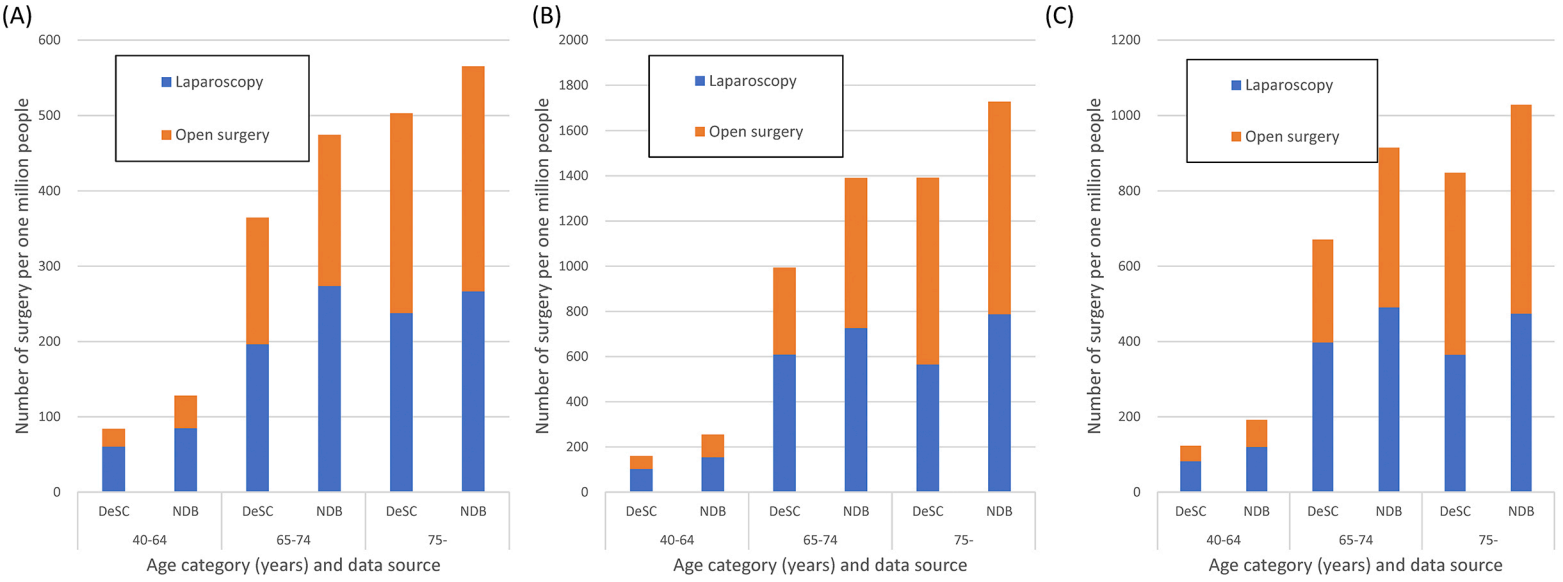
Comparison of proportion of patients undergoing gastric cancer stratified by age and sex between the DeSC database and NDB Open Data (A) Proportion of gastric surgery performed in women (B) Proportion of gastric surgery performed in men (C) Overall proportion of gastric surgery performed DeSC, the DeSC database; NDB, NDB Open Data.

In the DeSC database, the number of patients undergoing laparoscopic gastrectomy and open gastrectomy was 108 and 143, respectively, between December 2019 and November 2020, and the overall proportion of patients undergoing laparoscopic gastrectomy and open gastrectomy was 236 and 210 surgeries per million people, respectively. In comparison with the NDB Open Data between April 2019 and March 2020, the proportion of patients undergoing surgery is shown in Figure 6.

### Distribution of body mass index

We obtained 722,614 people (including 321,603 women and 401,011 men) who underwent health checkups in the DeSC database. The prevalence of body mass index in the overall, female, and male populations of the DeSC database and that of NHNSJ 2019 were similar (Supplementary Figure 2).

## Discussion

NDB can be regarded as the complete enumeration of the whole population in Japan, but commercially available administrative claims databases are based on nonrandom samples. Such databases can be used by many people; however, they should be carefully used in epidemiological and clinical studies. In the present study, we examined the population representativeness of the DeSC database, a newly developed commercially available administrative claims database. We calculated the prevalence of several NCDs including lifestyle-related diseases, cancer, and mental disorders. We confirmed that the distribution of age and sex in the DeSC data was comparable with that in the population estimates. The estimated prevalence of diabetes mellitus and hypertension was also comparable with that in the NHNS. The estimated prevalence of patients undergoing gastrectomy was also comparable with that of the NDB Open Data.

We found higher proportions in age categories of 65-69 and 70-74 years in the DeSC database compared with those among the entire Japanese population. This is because the proportions of participants in the insurance systems for self-employed and employees were different between the DeSC database and the whole Japanese population. In our study using the DeSC database, the numbers of participants in Kokuho and Kempo were approximately 1.1 million and 0.8 million, respectively, whereas the numbers of participants in Kokuho and the three employees’ insurance (Kempo, Kyokai Kempo, and Kyosai Kumiai) were 28.7 million and 77.0 million, respectively, in the whole population of Japan. That is, the DeSC database has a relatively high proportion of Kokuho participants. Most of those previously insured by the three employees’ insurance (Kempo, Kyokai Kempo, and Kyosai Kumiai) are to be insured by Kokuho from age 65 to 74 years, before participating in Koki Koreisha Iryo Seido. This may be because of the peak in 65-to-74-year-old people in the DeSC database.

The estimated prevalence of diabetes or hypertension in the DeSC database was similar to that reported in the NHNS. This may be attributed to the fact that the age distribution in the included population in the DeSC database was similar to that of Japan. Because the number of patients with diabetes reflects the number of those who consulted a physician for the disease, we found that the number of patients with diabetes in the DeSC database was almost the same as the number of patients with strongly suspected diabetes in the NHNS. Similarly, those with hypertension in the DeSC database were almost the same as those with hypertension on medication in the NHNS. Unlike other commercially available administrative databases in Japan, the DeSC database contains claims data collected by three types of insurers. Such a data structure is considered useful for improving the representation of the entire population in Japan.

The sex differences in the prevalence of osteoporosis, gastric cancer, and breast cancer are consistent with previous studies. A worldwide survey revealed that women are more susceptible to osteoporosis and therefore more prone to bone fractures ^(10)^. Regarding gastric cancer, a previous report showed consistent results with our results, confirming that all age groups of men were more likely to have gastric cancer than women ^(11)^. The proportion of male patients with breast cancer among all patients with breast cancer was 0.49%, which was similar to the proportion of male patients undergoing surgery for breast cancer among all patients undergoing surgery for breast cancer (0.58%) ^(12)^. This consistency suggests that even in diseases of low incidence, the DeSC data can yield the prevalence.

The proportions of patients undergoing gastrectomy in the DeSC database in comparison to those in the NDB Open Data were generally comparable. The period of data collection in the DeSC database and NDB Open Data was different, and considering the coronavirus pandemic, this gap may have affected the number of gastric surgeries.

This study has several limitations. First, although the DeSC database consists of three types of insurers, there are other types of insurers in Japan, which may still cause selection bias. Second, we calculated disease prevalence using the information on the basis of claims data, but there may be undiagnosed people or overdiagnosed people, which may have caused misclassification. Finally, we estimated the 1 year prevalence using claims data reimbursed from December 2019 to November 2020. Reportedly, the coronavirus pandemic may have kept patients away from physician visits ^(13)^, possibly resulting in an underestimation of the results.

In conclusion, we estimated the prevalence of several NCDs using the DeSC database. We confirmed the population representativeness of the database by comparing the population estimates and confirmed that the prevalence of diabetes mellitus and hypertension was comparable with that in a previously reported national survey. Our data using the new commercially available administrative claims database may be close to representing the entire population in Japan and can be utilized as basic information for policymaking in clinical medicine and public health in Japan.

## Article Information

### Conflicts of Interest

Akira Okada is a member of the Department of Prevention of Diabetes and Lifestyle-related Diseases, which is a cooperative program between the University of Tokyo and the Asahi Mutual Life Insurance Company.

### Author Contributions

AO performed the data collection and statistical analysis and wrote the first draft. HY performed the design of the research, revised the first draft, and supervised all the manuscript preparation. Both authors read and approved the final manuscript.

### Informed Consent

Because this study was a retrospective study using a commercial database, the need for informed consent was waived.

### Approval by Institutional Review Board (IRB)

This study protocol was approved by The Institutional Review Board of the Graduate School of Medicine of the University of Tokyo (2021010NI), and the study was conducted in accordance with the principles of the Helsinki Declaration.

## Supplement

Supplementary Materials
