# Supplementary material for: Prevalence of Noncommunicable Diseases in Japan Using a Newly Developed Administrative Claims Database Covering Young, Middle-aged, and Elderly People
Source: JMA J. 2022 Feb 28;5(2):190–8. doi: 10.31662/jmaj.2021-0189 (PMC9090547; doi:10.31662/jmaj.2021-0189)

Supplementary Table 1. Age distribution of those with checkup data available divided by the covered insurance

Age category (years)	Total<br>N=722,614	Kempo<br>N=412,320	Kokuho<br>N=261,777	Koki Koreisha Iryo Seido<br>N=48,517
15-19	0.5%	0.9%	0.0%	0.0%
20-24	2.8%	5.0%	0.0%	0.0%
25-29	4.6%	8.1%	0.0%	0.0%
30-34	4.9%	8.5%	0.0%	0.0%
35-39	6.9%	11.7%	0.5%	0.0%
40-44	12.5%	17.2%	7.4%	0.0%
45-49	11.3%	16.0%	6.1%	0.0%
50-54	10.1%	13.6%	6.4%	0.0%
55-59	7.6%	7.9%	8.5%	0.0%
60-64	10.9%	6.0%	20.5%	0.0%
65-69	17.2%	4.5%	40.2%	0.8%
70-74	4.1%	0.5%	10.4%	0.7%
75-79	4.3%	0.0%	0.0%	64.7%
80-84	1.6%	0.0%	0.0%	23.3%
85-89	0.6%	0.0%	0.0%	8.6%
90-94	0.1%	0.0%	0.0%	1.7%
95-99	0.0%	0.0%	0.0%	0.2%
100-	0.0%	0.0%	0.0%	0.0%

Supplementary Figure 1. Comparison of prevalence of depression and female breast cancer stratified by Kokuho and Kempo in the DeSC database

(A) Prevalence of depression

(B) Prevalence of female breast cancer

Supplementary Figure 1. Comparison of prevalence of depression and breast cancer stratified by Kokuho and Kempo in the DeSC database

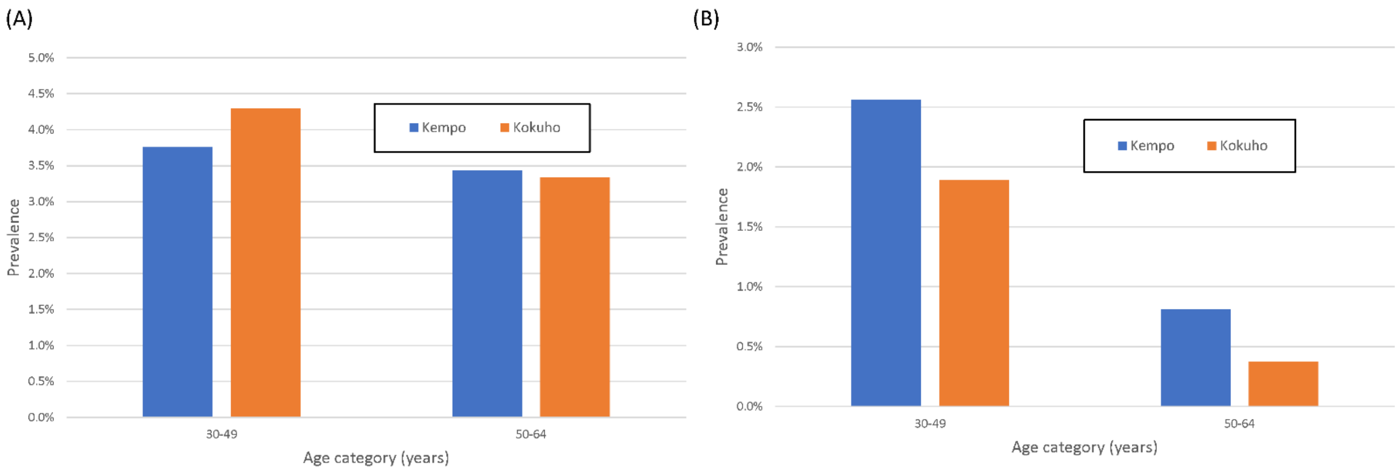

Supplementary Figure 2. Distribution of body mass index using DeSC database and National Health and Nutrition Survey 2019, stratified by sex and age category

(A) Distribution of body mass index in women

(B) Distribution of body mass index in men

(C) Overall distribution of body mass index in men

BMI, body mass index; DeSC, the DeSC database; NHNS, National Health and Nutrition Survey 2019

Supplementary Figure 2. Distribution of body mass index using DeSC database and National Health and Nutrition Survey 2019, stratified by sex and age category

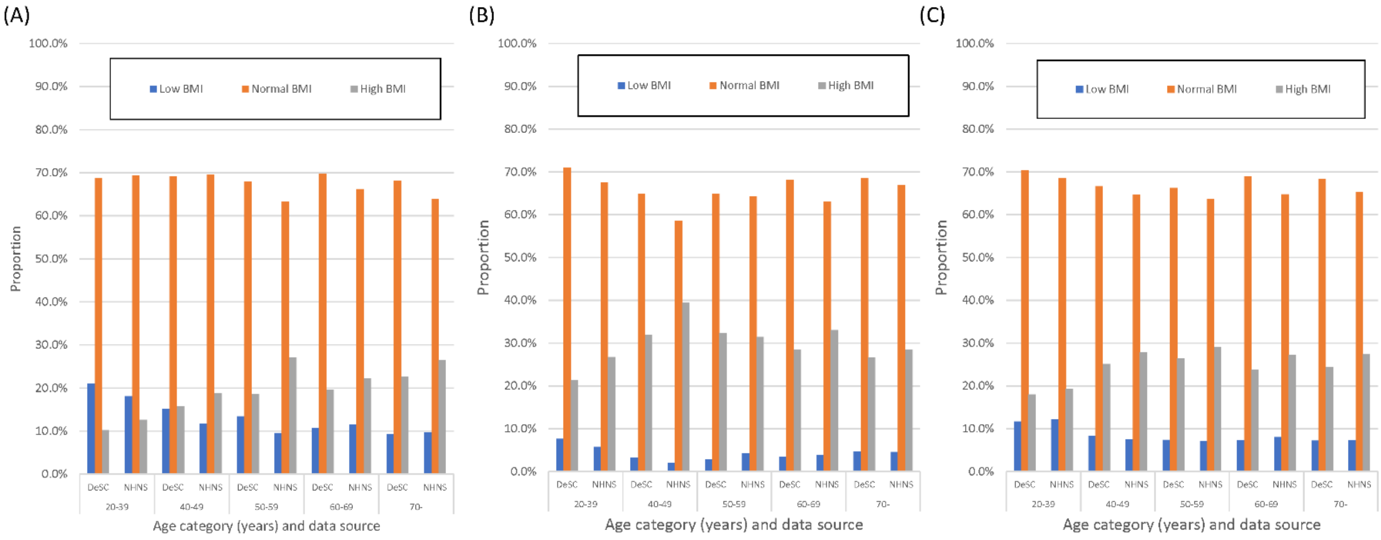

Supplement: Supplementary file 1 — Supplementary Materials [file 2433-3298-5-2-0190-s001.pdf]
